# Relationship between apolipoprotein M levels and diabetic retinopathy in patients with type 2 diabetes mellitus

**DOI:** 10.3389/fendo.2026.1809680

**Published:** 2026-04-13

**Authors:** Jin Ook Chung, Seon-Young Park, Dong Jin Chung, Min Young Chung

**Affiliations:** 1 Division of Endocrinology and Metabolism, Department of Internal Medicine, Chonnam National University Medical School, Gwangju, Republic of Korea; 2 Division of Gastroenterology and Hepatology, Department of Internal Medicine, Chonnam National University Medical School, Gwangju, Republic of Korea

**Keywords:** apolipoproteins, apolipoproteins M, diabetes mellitus, type 2, lipids, retinopathy, diabetic

## Abstract

Apolipoprotein M (apoM) is a biologically important protein that facilitates the mobilization and transport of cholesterol and other bioactive molecules in circulation. This study aims to explore the association between plasma apolipoprotein M (apoM) levels and diabetic retinopathy in patients with type 2 diabetes mellitus (T2DM). This cross−sectional study included 339 patients with T2DM. Patients with diabetic retinopathy exhibited greater median plasma apoM levels than those without diabetic retinopathy (26.05 [21.09–30.37] mg/L vs. 21.47 [18.00–26.38] mg/L; *p* < 0.001). In logistic regression models, plasma apoM levels were significantly associated with diabetic retinopathy (odds ratio [OR] per standard deviation increase in log_10_−transformed levels, 1.49; 95% confidence interval [CI], 1.05–2.119; *p* = 0.027) after adjusting for the confounders including age, hypertension, diabetes duration, and HbA_1c_. The area under the receiver operating characteristic curve was 0.657 (95% CI: 0.594–0.721), with internal bootstrap validation yielding a stable optimism-corrected area under the curve of 0.658. Our exploratory findings suggest a significant positive association between plasma apoM levels and diabetic retinopathy in patients with T2DM.

## Introduction

Diabetic retinopathy, a major microvascular complication of diabetes, frequently leads to visual impairment and blindness (1). Beyond its adverse effects on vision, diabetic retinopathy is also strongly associated with an increased risk of systemic vascular diseases, including stroke, heart failure, and coronary artery disease (2, 3). While hyperglycemia, hypertension, and diabetes duration are well-recognized risk factors for diabetic retinopathy, a comprehensive understanding of other risk factors remains limited (4). A growing body of evidence suggests that the pathogenesis of diabetic retinopathy is heterogeneous (5, 6).

The contribution of circulating lipids to the pathogenesis of diabetic retinopathy has been investigated in patients with diabetes. Studies show associations between low-density lipoprotein (LDL) cholesterol, triglycerides, and high-density lipoprotein (HDL) cholesterol and diabetic retinopathy in patients with type 2 diabetes mellitus (T2DM) (7–9), whereas other studies have failed to demonstrate similar relationships (10, 11). These inconsistent relationships between traditional serum lipids measures and diabetic retinopathy suggest that additional factors may contribute to its pathogenesis. Accordingly, apolipoproteins have gained interest because they may more accurately reflect lipid transport and vascular conditions than conventional serum lipid measurements.

Apolipoprotein M (apoM) is a biologically important protein that facilitates the mobilization and transport of cholesterol and other bioactive molecules in circulation. ApoM is crucial to the early stages of HDL metabolism, particularly in the formation of preβ-HDL particles (12). Although primarily associated with HDL, circulating apoM also binds apoB-containing lipoproteins (13). Additionally, apoM serves as a physiological carrier of the bioactive lipid sphingosine 1-phosphate (S1P) (14).

Previous studies report a relationship between apoM and cardiometabolic risk, highlighting its potential as a biomarker (15). Moreover, circulating apoM levels are closely associated with albuminuria and estimated glomerular filtration rate (eGFR) in patients with diabetes (16, 17). A recent study also reports that genetic variants in the promotor region of the APOM gene are associated with diabetic retinopathy (18). However, the association between circulating apoM levels and diabetic retinopathy in patients with T2DM remains unclear.

Therefore, this study aims to explore the relationship between plasma apoM levels and diabetic retinopathy in patients with T2DM.

## Methods

### Participants

This was a cross-sectional study conducted between August 2019 and January 2020. A total of 400 patients with T2DM attending the diabetes clinic at Chonnam National University Hospital were screened for eligibility. This study was conducted at Chonnam National University Hospital, a tertiary referral center in Gwangju, South Korea, which serves a diverse catchment population of approximately 1.2 million people. To minimize selection bias, we employed a consecutive enrollment strategy, approaching every patient who met the inclusion criteria during the study period.

**Figure 1:**
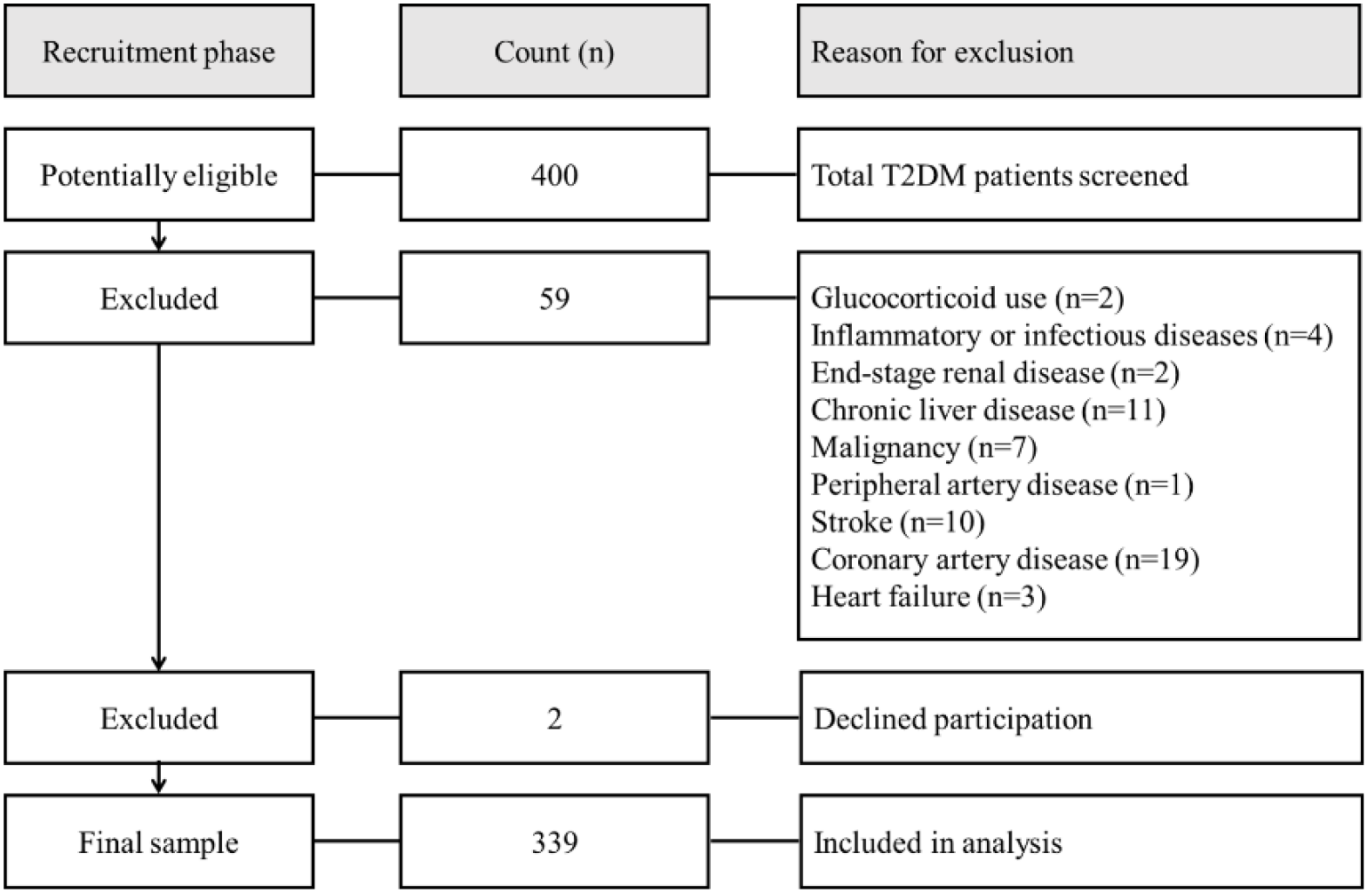
Flowchart of study cohort.

Of the screened population, 59 patients were excluded based on predefined criteria (**Figure 1**), including: glucocorticoid use, inflammatory or infectious diseases, end-stage renal disease, chronic liver disease, malignancy, peripheral artery disease, stroke, coronary artery disease, and heart failure. Additionally, 2 patients declined participation. Consequently, 339 patients were included in the final cross-sectional analysis. T2DM was diagnosed according to the American Diabetes Association’s ‘Standards of Medical Care in Diabetes’ (19). Hypertension was defined as blood pressure ≥ 140/90 mmHg or current use of antihypertensive medication. Hyperlipidemia was defined as total cholesterol level ≥6.5 mmol/L, triglyceride level ≥2.3 mmol/L, or current use of lipid-lowering therapy. The protocol was approved by an ethics committee of Chonnam National University Hospital (CNUH-2019-024), and informed consent was obtained from all participants.

### Measurements

Venous blood was collected after an overnight fast and centrifuged at 3,000 × *g* for 15 minutes. Aliquots were immediately stored at -80 °C and thawed only once, immediately prior to assaying, to minimize freeze-thaw cycles. All samples were processed in duplicate within a single laboratory to ensure technical uniformity. Glycated hemoglobin (HbA_1c_) levels were measured using ion-exchange liquid chromatography (Tosoh, Tokyo, Japan). Plasma apoM levels were quantified using an enzyme-linked immunosorbent assay kit (MyBioSource, San Diego, CA, USA) following the instructions of the manufacturer. Samples were diluted to fall within the linear detection range (1.56–100 mg/L), with a functional sensitivity of 0.39 mg/L. The inter-assay and intra-assay coefficients of variation were <12% and <10%, respectively.

eGFR was calculated using the Chronic Kidney Disease Epidemiology Collaboration equation (20). Urinary albumin excretion rate (ACR) was assessed from the urinary albumin-to-creatinine ratio in random urine samples. Diabetic retinopathy was evaluated by an ophthalmologist who remained masked to the patients’ clinical and laboratory status (including apoM levels) throughout the study. Following pupillary dilation, ultra-widefield fundus photography was performed on all participants using the Optos P2000Tx system (Optos PLC, Dunfermline, UK), which allows for a 200° internal angle view of the retina in a single capture. These fundus photographs served as the primary basis for grading, which followed the International Clinical Diabetic Retinopathy (ICDR) disease severity scale (21). Participants were categorized into three groups based on the more severely affected eye: (1) no diabetic retinopathy: no retinal abnormalities (ICDR level 1); (2) nonproliferative diabetic retinopathy: presence of at least one microaneurysm, intraretinal hemorrhage, hard exudate, or venous beading, without evidence of neovascularization (ICDR levels 2–4); and (3) proliferative diabetic retinopathy: presence of neovascularization, vitreous hemorrhage, or preretinal hemorrhage (ICDR level 5). To ensure the robustness of the data, a random subset of 60 photographs (18% of the total sample) was re-graded by the same ophthalmologist, demonstrating good intra-grader reliability (Kappa = 0.90).

Given the cross-sectional design of the study, plasma apoM levels and diabetic retinopathy were assessed simultaneously; therefore, the observed associations do not imply a temporal sequence or causal relationship.

### Statistical analyses

A *post hoc* power analysis was performed to evaluate the adequacy of the sample size (n=339) for detecting the observed association between apoM levels and retinopathy. The analysis was conducted using G*Power (3.1.9.2) for logistic regression, assuming a two-tailed test with an alpha level of 0.05. To account for the complexity of the adjusted models, the calculation incorporated a range of R-squared values (*R*^2^ = 0.1 to 0.2) representing the variance in apoM explained by other covariates in the model. With a total sample size of 339 participants and an observed retinopathy prevalence of 31.0%, the study achieved a *post hoc* statistical power of 88.1% (assuming *R*^2^ for other covariates = 0.1) and 84.0% (assuming *R*^2^ for other covariates = 0.2) to detect the reported adjusted Odds Ratio for apoM. These values exceed the standard 80% power threshold, suggesting that the sample size was sufficient to provide a reliable estimate of the association, after adjusting for potential confounders.

Continuous variables were assessed for normality using the Kolmogorov-Smirnov test. Data are presented as mean ± standard deviation (SD) for normally distributed variables, median (interquartile range) for skewed variables, or frequency (percentage) for categorical data. Categorical variables were analyzed with the chi-squared test or Fisher’s exact test for two-group comparisons, while continuous variables were compared using the Student’s t-test or Mann–Whitney U test. Differences among the apoM tertile groups were assessed using the Kruskal–Wallis test, Fisher’s exact test, or analysis of variance. Comparisons across diabetic retinopathy stages were performed using the Kruskal-Wallis test, followed by Dunn’s *post-hoc* test with Bonferroni correction for pairwise comparisons to identify specific differences between disease severities.

Multivariable logistic regression analysis was performed using the enter method to evaluate the independent association between plasma apoM levels and diabetic retinopathy. To ensure a parsimonious model and address potential overfitting, covariates were selected through a combination of clinical relevance and statistical screening: (1) pre-specified clinical confounders (age, HbA_1c_, and diabetes duration) were included regardless of their statistical significance (4), and (2) potential confounders demonstrating a univariate association of *p* < 0.10 were added, including hypertension, high-sensitivity C-reactive protein (hs-CRP), HDL-cholesterol, urinary ACR, eGFR, and use of insulin or oral hypoglycemic agents (OHAs) (**Supplementary Table 1**). Although apoA-I differed between groups in rank-based testing (*p* = 0.014), it was excluded from the multivariable model due to a lack of univariable logistic association (*p* = 0.497) and to maintain model stability.

We employed a hierarchical adjustment strategy across three incremental models. Model 1: Adjusted for age, hypertension, hs-CRP, and HDL-cholesterol. Model 2: Further adjusted for diabetes duration, HbA_1c_, and medication use (OHAs and insulin). Model 3: Additionally incorporated markers of renal function (eGFR and urinary ACR) to test if the apoM association remained independent of generalized microvascular impairment, while acknowledging the potential for overadjustment in this final step. Variables with skewed distributions were log_10_-transformed before analysis. Odds ratios (ORs) per 1-SD increase in log_10_-transformed apoM levels were estimated. ApoM was analyzed both as a continuous variable (per 1-SD increase in log_10_-transformed levels) and in tertiles, with a *p* for trend calculated by entering the tertiles as an ordinal variable to assess for a dose-response relationship. No significant interactions were observed between apoM and the included covariates (*p* for interaction > 0.05).Given the exploratory nature of this study, several steps were taken to ensure model stability: (1) power and overfitting: with 105 observed cases of diabetic retinopathy and 11 independent variables, the events per variable (EPV) ratio was approximately 9.6, providing a balance between confounding control and sample size constraints, (2) collinearity: variance inflation factors (VIF) were remained below 2.5, indicating no significant multicollinearity (**Supplementary Table 2**), (3) medication bias: insulin and OHA use were included as dummy variables serving as clinical proxies for disease severity, and (4) renal confounding: A sensitivity analysis was performed by excluding patients with overt macroalbuminuria (>300 mg/g) or severely reduced eGFR (<30 ml min^-1^1.73m^-2^) to ensure the association was independent of severe renal disease.

The discriminative ability of apoM was assessed using receiver operating characteristic (ROC) curves. The area under the curve (AUC), sensitivity, and specificity were calculated, with the optimal cut-off determined by the Youden index. Internal validation was performed using 1,000 bootstrap iterations to calculate bias-corrected and accelerated (BCa) 95% confidence intervals, which are robust to model optimism and distributional assumptions. Due to rigorous data collection, there was 0% missingness for all key variables. No formal adjustment for multiple comparisons was applied given the exploratory nature of the study; instead, we prioritized the interpretability of effect sizes and biological plausibility. Statistical significance was set as *p* < 0.05. Bootstrapping was performed using R software, while all other analyses were conducted using SPSS version 20.0.

## Results

**Table 1** presents the clinical characteristics of patients with T2DM. Compared with those without diabetic retinopathy, patients with diabetic retinopathy had longer diabetes duration, higher systolic blood pressure and HbA_1c_, lower HDL-cholesterol level and apoA-I level, higher urinary ACR, lower eGFR, and a higher prevalence of hypertension, OHA use, and insulin therapy. Plasma apoM levels were significantly higher in patients with diabetic retinopathy than in those without. Plasma apoM levels differed significantly across diabetic retinopathy stages (21.47 [18.00–26.38] mg/L for no retinopathy [n = 234], 25.79 [20.90–29.81] mg/L for nonproliferative retinopathy [n = 77], and 26.91 [21.81–30.55] mg/L for proliferative retinopathy [n = 28]; *p* < 0.001). However, no statistically significant difference was observed between nonproliferative and proliferative groups (*p* = 0.562).

**Table 1:** Characteristics of individuals with type 2 diabetes mellitus based on diabetic retinopathy.

Variables	Diabetic retinopathy (–)	Diabetic retinopathy (+)	*p*-value
n	234	105	
Age (years)	59.9 ± 14.3	60.7 ± 11.5	0.639
Men (%)	120 (51.3)	53 (50.5)	0.891
Diabetes duration (years)	4.0 (0.3–10.0)	14.0 (5.5–20.0)	<0.001
BMI (kg/m^2^)	25.7 ± 4.4	25.3 ± 4.1	0.510
Hyperlipidemia, n (%)	192 (82.1)	86 (81.9)	0.974
Hypertension, n (%)	136 (58.1)	76 (72.4)	0.012
Systolic BP (mmHg)	132.8 ± 16.8	136.9 ± 15.0	0.033
Diastolic BP (mmHg)	77.3 ± 11.8	76.4 ± 10.9	0.501
HbA_1c_ (mmol/mol) (%)	61 ± 18 (7.7 ± 1.6)	71 ± 18 (8.6 ± 1.7)	<0.001
Total cholesterol (mmol/l)	4.1 (3.4–4.9)	3.7 (3.2–4.8)	0.053
Triglyceride (mmol/l)	1.3 (0.9–2.0)	1.3 (1.0–2.1)	0.286
HDL-cholesterol (mmol/l)	1.3 (1.1–1.5)	1.2 (1.0–1.4)	0.005
LDL-cholesterol (mmol/l)	2.3 (1.8–3.0)	2.0 (1.7–2.8)	0.088
hs-CRP (mg/L)	0.6 (0.3–1.4)	0.7 (0.3–1.4)	0.591
ApoM (mg/L)	21.47 (18.00–26.38)	26.05 (21.09–30.37)	<0.001
ApoA-I (g/L)	1.38 (1.23–1.47)	1.31 (1.16–1.46)	0.014
ApoB (g/L)	0.82 (0.68–0.98)	0.76 (0.65–0.93)	0.355
eGFR (ml min^-1^1.73m^-2^)	89.8 ± 21.9	83.7 ± 23.2	0.020
Urinary ACR (mg/g)	11.5 (6.8–27.6)	35.8 (15.5–143.9)	<0.001
OHAs, n (%)	176 (75.2)	90 (85.7)	0.030
Sulfonylurea, n (%)	57 (24.4)	43 (41.0)	0.002
Metformin, n (%)	165 (70.5)	80 (76.2)	0.280
DPP-4*i*, n (%)	114 (78.7)	63 (60.0)	0.054
TZD, n (%)	22 (9.4)	9 (8.6)	0.806
SGLT2*i*, n (%)	25 (10.7)	12 (11.4)	0.839
GLP1-RA, n (%)	0 (0.0)	2 (1.9)	N/A
Insulin, n (%)	19 (8.1)	45 (42.9)	<0.001
Lipid-lowering agents, n (%)	181 (77.4)	78 (74.3)	0.539
Statin, n (%)	177 (75.6)	78 (74.3)	0.789
Fibrate, n (%)	11 (4.7)	3 (2.9)	0.562
Ezetimibe, n (%)	26 (11.1)	16 (15.2)	0.286

Data are presented as mean or percent (SD), or median (interquartile range) unless stated otherwise. HbA_1c_ data are presented as IFCC units (mmol/mol) and NGSP units (%). BMI, body mass index; BP, blood pressure; HbA_1c_, glycated hemoglobin; NGSP, National Glycohemoglobin Standardization Program; IFCC, International Federation of Clinical Chemistry; HDL-cholesterol, high density lipoprotein cholesterol; LDL-cholesterol, low density lipoprotein cholesterol; hs-CRP, high-sensitivity C-reactive protein; apoM, apolipoprotein M; apoA-I, apolipoprotein A-I; apoB, apolipoprotein B; eGFR, estimated glomerular filtration rate; ACR, albumin excretion rate; OHAs, oral hypoglycemic agents; DPP-4*i*, dipeptidyl peptidase-4 inhibitor; TZD, thiazolidinediones; SGLT2*i*, sodium-glucose transport 2 inhibitor; GLP1-RA, glucagon-like peptide-1 receptor agonist.

**Table 2** shows the patient characteristics based on plasma apoM tertiles. Men, diabetes duration, urinary ACR, and the prevalence of hyperlipidemia, OHA use, insulin use, and lipid-lowering therapy increased across apoM tertiles. The prevalence of diabetic retinopathy also rose with higher apoM tertiles. Additionally, BMI and eGFR differed significantly across apoM tertiles (**Table 2**).

**Table 2:** Characteristics of patients with T2DM based on plasma apoM tertiles.

Variables	ApoM (mg/L)			*p*-value
	Tertile1	Tertile2	Tertile3	
n	113	113	113	
Age (years)	58.4 ± 15.3	61.1 ± 13.2	60.9 ± 11.7	0.252
Men (%)	43 (38.1)	57 (50.4)	73 (64.6)	<0.001
Diabetes duration (years)	3.0 (0.2–8.0)	7.0 (0.8–12.0)	12.5 (4.5–20.0)	<0.001
BMI (kg/m^2^)	25.7 ± 3.9	26.2 ± 4.9	24.7 ± 4.0	0.033
Hyperlipidemia, n (%)	82 (72.6)	94 (83.2)	102 (90.3)	0.002
Hypertension, n (%)	64 (56.6)	70 (61.9)	78 (69.0)	0.155
Systolic BP (mmHg)	133.3 ± 17.3	133.6 ± 16.3	135.5 ± 15.6	0.536
Diastolic BP (mmHg)	77.6 ± 11.6	76.4 ± 10.8	77.1 ± 12.1	0.727
HbA_1c_ (mmol/mol) (%)	62 ± 18 (7.8 ± 1.6)	63 ± 18 (7.9 ± 1.7)	67 ± 19 (8.3 ± 1.7)	0.063
Total cholesterol (mmol/l)	4.0 (3.5–5.0)	4.0 (3.4–4.8)	3.9 (3.1–4.9)	0.493
Triglyceride (mmol/l)	1.3 (1.0–2.1)	1.3 (0.9–2.0)	1.3 (1.0–2.1)	0.597
HDL-cholesterol (mmol/l)	1.3 (1.1–1.5)	1.3 (1.1–1.5)	1.2 (1.1–1.4)	0.637
LDL-cholesterol (mmol/l)	2.3 (1.9–3.1)	2.2 (1.8–2.8)	2.1 (1.6–2.9)	0.159
hs-CRP (mg/L)	0.7 (0.3–1.5)	0.7 (0.2–1.6)	0.5 (0.3–1.1)	0.147
ApoM (mg/L)	16.86 (14.44–18.75)	22.73 (21.22–24.64)	30.67 (28.40–34.49)	<0.001
ApoA-I (g/L)	1.35 (1.17–1.46)	1.36 (1.21–1.47)	1.38 (1.23–1.50)	0.233
ApoB (g/L)	0.82 (0.67–1.00)	0.83 (0.68–0.95)	0.75 (0.65–0.92)	0.182
Diabetic retinopathy, n (%)	20 (17.7)	33 (29.2)	52 (46.0)	<0.001
eGFR (ml min^-1^1.73m^-2^)	96.9 ± 17.3	98.4 ± 20.9	78.6 ± 24.9	<0.001
Urinary ACR (mg/g)	11.5 (6.7–24.9)	15.5 (7.4–35.1)	29.6 (9.9–148.0)	<0.001
OHAs, n (%)	82 (72.6)	87 (77.0)	97 (85.8)	0.047
Sulfonylurea, n (%)	27 (23.9)	38 (33.6)	35 (31.0)	0.253
Metformin, n (%)	76 (67.3)	81 (71.7)	88 (77.9)	0.201
DPP-4*i*, n (%)	53 (46.9)	61 (54.0)	63 (55.8)	0.370
TZD, n (%)	5 (4.4)	14 (12.4)	12 (10.6)	0.093
SGLT2*i*, n (%)	12 (10.6)	8 (7.1)	17 (15.0)	0.157
GLP1-RA, n (%)	0 (0.0)	1 (0.9)	1 (0.9)	N/A
Insulin, n (%)	15 (13.3)	16 (14.2)	33 (29.2)	0.003
Lipid-lowering agent, n (%)	75 (66.4)	87 (77.0)	97 (85.8)	0.003
Statin, n (%)	74 (65.5)	84 (74.3)	97 (85.8)	0.002
Fibrate, n (%)	2 (1.8)	4 (3.5)	8 (7.1)	0.156
Ezetimibe, n (%)	13 (11.5)	12 (10.6)	17 (15.0)	0.565

Data are expressed as mean or percent (SD), or median (interquartile range) unless stated otherwise. HbA_1c_ data are presented as IFCC units (mmol/mol) and NGSP units (%). BMI, body mass index; BP, blood pressure; HbA_1c_, glycated hemoglobin; HDL-cholesterol, high density lipoprotein cholesterol; LDL-cholesterol, low density lipoprotein cholesterol; hs-CRP, high-sensitivity C-reactive protein; apoM, apolipoprotein M; apoA-I, apolipoprotein A-I; apoB, apolipoprotein B; eGFR, estimated glomerular filtration rate; ACR, albumin excretion rate; OHAs, oral hypoglycemic agents; DPP-4*i*, dipeptidyl peptidase-4 inhibitor; TZD, thiazolidinediones; SGLT2*i*, sodium-glucose transport 2 inhibitor; GLP1-RA, glucagon-like peptide-1 receptor agonist.

Logistic regression was used to evaluate the independent association between plasma apoM levels and diabetic retinopathy (**Table 3**). In Model 2, which adjusted for age, hs-CRP, hypertension, HDL-cholesterol, HbA_1c_, diabetes duration, and use of anti-hyperglycemic agents, apoM was significantly associated with retinopathy (OR per 1-SD increase in log_10_-transformed levels = 1.45; 95% confidence interval (CI): 1.07–1.95; *p* = 0.016). To test if this link was merely a reflection of systemic microvascular dysfunction, Model 3 further adjusted for renal markers (eGFR and urinary ACR). The point estimate remained stable and shifted slightly away from the null (OR = 1.49, 95% CI, 1.05–2.11; *p* = 0.027), suggesting a distinct pathway independent of renal impairment. This relationship also revealed a significant dose-response pattern across tertiles (*p* for trend = 0.038), with the highest tertile showing a two-fold increase in risk (OR 2.03; 95% CI: 1.10–3.76) compared to the lowest.

**Table 3:** Logistic regression models of the association between apoM levels and diabetic retinopathy in patients with T2DM.

Variable	Unadjusted model		Model 1		Model 2		Model 3	
	OR (95% CI)	*p*-value	OR (95% CI)	*p*-value	OR (95% CI)	*p*-value	OR (95% CI)	*p*-value
ApoM (mg/L)^†^	1.72 (1.34–2.23)	<0.001	1.85 (1.41–2.41)	<0.001	1.45 (1.07–1.95)	0.016	1.49 (1.05–2.11)	0.027
Tertile 1	1.00 (reference)		1.00 (reference)		1.00 (reference)		1.00 (reference)	
Tertile 2	1.92 (1.02–3.60)		1.91 (1.04–3.51)		1.66 (0.65-4.20)		1.62 (0.60–4.34)	
Tertile 3	1.99 (1.47–2.70)		2.29 (1.54–3.40)		1.99 (1.15–3.45)		2.03 (1.10–3.76)	
*P* for trend	<0.001		<0.001		0.026		0.038	

^†^Data were log_10_-transformed before analysis.

Model 1: adjusted based on age, hypertension, hs-CRP^†^, and HDL-cholesterol^†^.

Model 2: adjusted by model 1 plus HbA_1c_, and diabetes duration^†^, and use of OHAs and insulin.

Model 3: adjusted by model 2 plus urinary ACR^†^ and eGFR.

apoM, apolipoprotein M; HbA_1c_, glycated hemoglobin; HDL, high-density lipoprotein; hs-CRP, high-sensitivity C-reactive protein; eGFR, estimated glomerular filtration rate; ACR, albumin excretion rate; OHAs, oral hypoglycemic agents.

Sensitivity analyses confirmed these findings. When hyperlipidemia was substituted for HDL-cholesterol, the association remained significantly (*p* = 0.031) (**Supplementary Table 3**). Adjusting for anti-hyperglycemic and lipid-lowering therapy resulted in minimal point estimates shift (4.9% and 2.8%, respectively) (**Supplementary Table 4**). Furthermore, in a subgroup excluding patients with overt macroalbuminuria or severely reduced eGFR (n = 308), the association persisted (OR = 1.54, 95% CI, 1.07-2.23, *p* = 0.021). Collinearity diagnostics showed Variance Inflation Factors (VIF) below 2.5, ensuring model stability (**Supplementary Table 2**).

**Figure 2:**
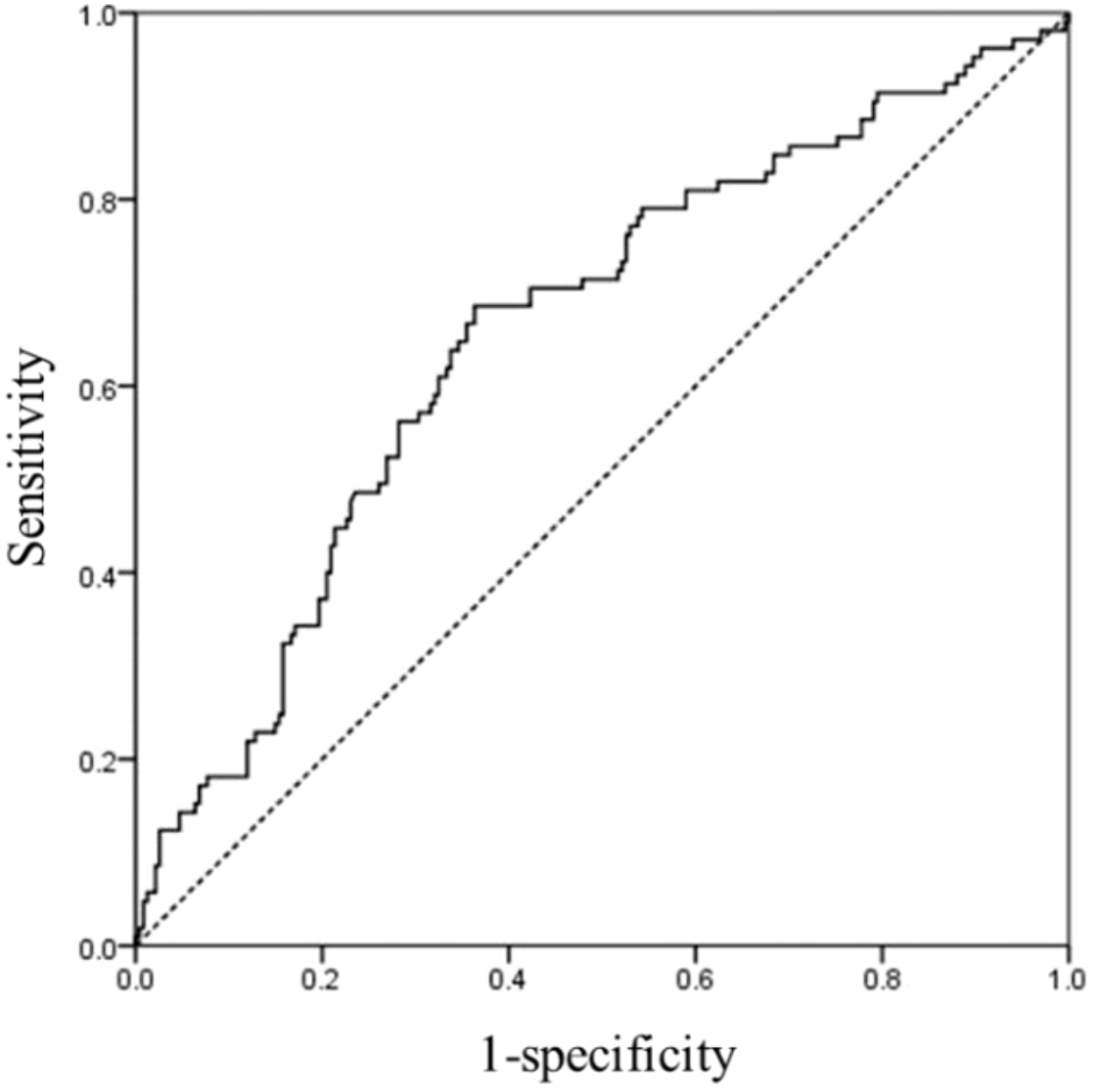
ROC curve for plasma apoM in patients with T2DM. AUC = 0.657 (*p* < 0.001), 95% CI: 0.594–0.721. ApoM cutoff = 23.59 mg/L; sensitivity: 68.6%; specificity: 63.7%. The 95% CI was further validated by 1,000 bootstrap resamples (BCa 95% CI: 0.594–0.721), confirming model stability. ROC, receiver operating characteristic; apoM, apolipoprotein M; AUC, area under the curve; T2DM, type 2 diabetes mellitus.

**Figure 2** illustrates the ROC curve analysis for apoM, yielding an AUC of 0.657 (95% CI: 0.594–0.721; *p* < 0.001), indicating a modest level of discrimination for diabetic retinopathy. Upon internal validation with 1,000 bootstrap resamples, the optimism-corrected AUC was 0.658 (95% bootstrap CI: 0.594–0.721). The fact that the bootstrap interval is nearly identical to the original interval confirms high model precision and suggests that the findings are not driven by specific outliers, indicating a robust and reproducible association within the study population.

## Discussion

This exploratory study showed that plasma apoM levels were positively associated with diabetic retinopathy in patients with T2DM, suggesting that apoM may serve as a biological marker associated with the presence of diabetic retinopathy.

ApoM, an HDL-associated apolipoprotein, is involved in diverse biological functions, including lipoprotein metabolism, endothelial integrity, and inflammatory processes (22). Given that these functions are closely linked to diabetes-related vasculopathy (4), apoM may be implicated in the pathogenesis of diabetic retinopathy. However, the association between plasma apoM levels and diabetic retinopathy in patients with T2DM remains unclear. In this study, plasma apoM levels were higher in patients with diabetic retinopathy than in those without. Significant positive associations between apoM levels and diabetic retinopathy persisted after adjusting for age, hypertension, hs-CRP, and HDL-cholesterol (Model 1); HbA_1c_, diabetes duration, and use of anti-hyperglycemic agents (Model 2); and urinary ACR and eGFR (Model 3).

In this study, plasma apoM levels were associated with diabetes duration, a surrogate marker of cumulative glycemic exposure, as well as with dyslipidemia and renal function. Consistent with our findings, previous studies report that plasma apoM levels are associated with glycemic status and lipoprotein cholesterol content (23, 24). ApoM levels in the circulation are also linked to albuminuria and eGFR in patients with diabetes (16, 17). Thus, these factors may partially explain the association between apoM and diabetic retinopathy observed in the present study, as they are also involved in diabetic retinopathy (4). However, this relationship remained significant after multivariable adjustment, suggesting that it is not significantly influenced by several key clinical confounders measured in this study. However, we cannot exclude the possibility of residual confounding from unmeasured factors that may have influenced these results.

The observed positive association between apoM levels and diabetic retinopathy was unexpected, given apoM’s known vasculoprotective roles (14). While the precise biological role of apoM in the pathogenesis of diabetic retinopathy remains to be elucidated, we hypothesize that elevated apoM levels observed in our cohort may reflect a compensatory response to vascular stress. In this speculative model, the upregulation of apoM could represent a physiological attempt to stabilize a compromised blood-retinal barrier. However, as our study is cross-sectional, this interpretation remains speculative and necessitates further mechanistic validation in translational or *in vitro* models. Additional factors may also be considered. Besides an association with HDL, apoM binds apoB-containing lipoproteins and exchanges between other lipoproteins such as VLDL, LDL, and chylomicrons (13, 25). An experimental study shows that mice overexpressing human apoM exhibit increased cholesterol, triglyceride, and S1P levels (26). In humans, increased apoM levels are associated with higher LDL-cholesterol in addition to HDL-cholesterol (24). Stadler et al. report that low plasma apoM levels are associated with an increased risk of adverse cardiovascular events in patients with chronic kidney disease (27). In contrast, Ahnström et al. show no association between plasma apoM levels and coronary heart disease (28). Zhang et al. (17) report higher plasma apoM levels in patients with T2DM associated with diabetic nephropathy than in those without. Baker et al. also report that higher apoM is associated with increased risk of kidney dysfunction progression in patients with T1DM (16). Thus, apoM may be associated with other biological pathways, potentially counteracting its benefits via HDL interactions. In diabetes, HDL can be glycated (29), potentially reducing the binding capacity of apoM to S1P (30). Finally, apoM can bind small ligands, including fatty acids, oxidized phospholipids, and S1P (31, 32). However, despite these hypotheses, the directionality of the relationship remains unverified within the constraints of this cross-sectional analysis. Furthermore, as exposure and outcome were measured simultaneously, our findings support a biological association rather than a causal link, and reverse causation remains a plausible explanation. Further longitudinal studies are needed to explore the causality and mechanisms between apoM and diabetic retinopathy in patients with T2DM.

Our data indicated that apoM levels were comparable between nonproliferative and proliferative retinopathy subgroups. This suggests that while apoM is associated with the presence of retinopathy, it may not linearly correlate with increasing clinical severity. Consequently, we assume that apoM might be particularly relevant to the early development of diabetic retinopathy; however, this requires confirmation in large-scale longitudinal studies.

Several limitations must be acknowledged. First, the cross-sectional design precludes the establishment of a temporal sequence or causal relationship between plasma apoM levels and diabetic retinopathy. While our findings suggest apoM is a robust marker, longitudinal studies are required to verify its predictive value over time. Second, our study was conducted at a single tertiary center with stringent exclusion criteria, specifically omitting patients with overt macrovascular disease and significant comorbidities. While this approach enhanced internal validity by reducing noise from these comorbidities and systemic inflammation, it may introduce selection bias. In addition, our cohort may represent a specific subset of the T2DM population, and our results might overestimate the association strength compared to patients in primary care or those with a higher systemic disease burden. Third, the sample size and event rate (n= 105 for diabetic retinopathy), while meeting the requirements for stable modeling, may limit the statistical power for extensive subgroup analyses. The borderline significance in some adjusted models suggests that our results should be viewed as hypothesis-generating. Furthermore, the risk of Type I error due to multiplicity in subgroup and tertile comparisons should be noted; thus, these results require validation in larger, independent cohorts. Fourth, regarding medication data, we utilized binary indicators for treatment. Although the conceptual framing of medications as markers of disease severity was supported by sensitivity analyses, the lack of granular data on treatment duration and intensity may result in residual confounding. We acknowledge that insulin use, while serving as a surrogate for glycemic volatility and beta-cell exhaustion, is a simplification of complex long-term metabolic control. Finally, the AUC of 0.657 indicates modest discriminative ability. While internal bootstrap validation confirmed the stability of this marker, apoM should be viewed as a potential component of a multi-marker risk profile rather than a standalone diagnostic tool. Future research should explore whether combining apoM with other novel biomarkers or imaging metrics can enhance the accuracy of retinopathy screening in clinical practice.

In conclusion, this exploratory study showed that plasma apoM levels were positively associated with diabetic retinopathy in patients with T2DM. Future prospective longitudinal studies and external validation in diverse clinical settings are required to determine whether apoM can serve as a reliable clinical marker for the development or progression of diabetic retinopathy.

## Data Availability

The original contributions presented in the study are included in the article/**Supplementary Material**. Further inquiries can be directed to the corresponding author.

## References

[1] American Diabetes Association Professional Practice Committee . 12. Retinopathy, neuropathy, and foot care: Standards of care in diabetes-2025. Diabetes Care. (2025) 48:S252–65. doi: 10.2337/dc25-S012, PMID: 39651973 PMC11635040

[2] Wong KH Hu K Peterson C Sheibani N Tsivgoulis G Majersik JJ . Diabetic retinopathy and risk of stroke: A secondary analysis of the ACCORD Eye Study. Stroke. (2020) 51:3733–6. doi: 10.1201/9781315146737-16 PMC7686117 33019896

[3] Modjtahedi BS Wu J Luong TQ Gandhi NK Fong DS Chen W . Severity of diabetic retinopathy and the risk of future cerebrovascular disease, cardiovascular disease, and all-cause mortality. Ophthalmology. (2021) 128:1169–79. doi: 10.1016/j.ophtha.2020.12.019. PMID: 33359888

[4] Cheung N Mitchell P Wong TY . Diabetic retinopathy. Lancet. (2010) 376:124–36. doi: 10.1016/j.ophtha.2007.07.010. PMID: 20580421

[5] Pedersen FN Stokholm L Andersen N Andresen J Bek T Hajari J . Risk of diabetic retinopathy according to subtype of type 2 diabetes. Diabetes. (2024) 73:977–82. doi: 10.2337/figshare.25407979. PMID: 38498373 PMC11109772

[6] Luk AOY Fan Y Fan B Chow EWK O TCK . Heterogeneity in the development of diabetes-related complications: Narrative review of the roles of ancestry and geographical determinants. Diabetologia. (2025) 68:2386–404. doi: 10.1007/s00125-025-06482-8. PMID: 40696182 PMC12534336

[7] Rema M Srivastava BK Anitha B Deepa R Mohan V . Association of serum lipids with diabetic retinopathy in urban South Indians--the Chennai Urban Rural Epidemiology Study (CURES) Eye Study--2. Diabetes Med. (2006) 23:1029–36. doi: 10.1111/j.1464-5491.2006.01890.x. PMID: 16922712

[8] Sasso FC Pafundi PC Gelso A Bono V Costagliola C Marfella R . High HDL cholesterol: A risk factor for diabetic retinopathy? Findings from NO BLIND study. Diabetes Res Clin Pract. (2019) 150:236–44. doi: 10.1016/j.diabres.2019.03.028. PMID: 30904748

[9] Tan GS Gan A Sabanayagam C Tham YC Neelam K Mitchell P . Ethnic differences in the prevalence and risk factors of diabetic retinopathy: The Singapore Epidemiology of Eye Diseases Study. Ophthalmology. (2018) 125:529–36. doi: 10.1016/j.ophtha.2017.10.026. PMID: 29217148

[10] Mohamed Q Gillies MC Wong TY . Management of diabetic retinopathy: A systematic review. JAMA. (2007) 298:902–16. doi: 10.1093/oso/9780195340235.003.0014. PMID: 17712074

[11] Klein BE Moss SE Klein R Surawicz TS . The Wisconsin Epidemiologic Study of Diabetic Retinopathy. XIII. Relationship of serum cholesterol to retinopathy and hard exudate. Ophthalmology. (1991) 98:1261–5. doi: 10.1001/archopht.1994.01090210105023. PMID: 1923364

[12] Wolfrum C Poy MN Stoffel M . Apolipoprotein M is required for prebeta-HDL formation and cholesterol efflux to HDL and protects against atherosclerosis. Nat Med. (2005) 11:418–22. doi: 10.1038/nm1211. PMID: 15793583

[13] Christoffersen C Nielsen LB Axler O Andersson A Johnsen AH Dahlback B . Isolation and characterization of human apolipoprotein M-containing lipoproteins. J Lipid Res. (2006) 47:1833–43. doi: 10.1194/jlr.m600055-jlr200. PMID: 16682745

[14] Christoffersen C Obinata H Kumaraswamy SB Galvani S Ahnstrom J Sevvana M . Endothelium-protective sphingosine-1-phosphate provided by HDL-associated apolipoprotein M. Proc Natl Acad Sci USA. (2011) 108:9613–8. doi: 10.1073/pnas.1103187108. PMID: 21606363 PMC3111292

[15] Christoffersen C . Apolipoprotein M-a marker or an active player in type II diabetes? Front Endocrinol (Lausanne). (2021) 12:665393. doi: 10.3389/fendo.2021.665393. PMID: 34093440 PMC8176018

[16] Baker NL Hammad SM Hunt KJ Semler A Klein RL Lopes-Virella MF . Plasma apoM levels and progression to kidney dysfunction in patients with type 1 diabetes. Diabetes. (2022) 71:1795–9. doi: 10.2337/db21-0920. PMID: 35554520 PMC9490352

[17] Zhang P Gao J Pu C Feng G Wang L Huang L . ApoM/HDL-C and apoM/apoA-I ratios are indicators of diabetic nephropathy in healthy controls and type 2 diabetes mellitus. Clin Chim Acta. (2017) 466:31–7. doi: 10.1016/j.cca.2017.01.006. PMID: 28073663

[18] Tageldeen MM Badrawy H Abdelmeguid M Zaghlol M Gaber N Kenawy EM . Apolipoprotein M gene polymorphism Rs805297 (C-1065A): Association with type 2 diabetes mellitus and related microvascular complications in South Egypt. Am J Med Sci. (2021) 362:48–55. doi: 10.1016/j.amjms.2021.02.002. PMID: 33621527

[19] American Diabetes Association . 2. Classification and diagnosis of diabetes: Standards of medical care in diabetes-2019. Diabetes Care. (2019) 42:S13–28. doi: 10.2337/diacare.27.2007.s15. PMID: 30559228

[20] Levey AS Stevens LA Schmid CH Zhang YL Castro A Feldman HI . A new equation to estimate glomerular filtration rate. Ann Intern Med. (2009) 150:604–12. doi: 10.7326/0003-4819-150-9-200905050-00006. PMID: 19414839 PMC2763564

[21] Wilkinson CP Ferris F Klein RE Lee PP Agardh CD Davis M . Proposed international clinical diabetic retinopathy and diabetic macular edema disease severity scales. Ophthalmology. (2003) 110:1677–82. doi: 10.1016/s0161-6420(03)00475-5. PMID: 13129861

[22] Diarte-Anazco EMG Mendez-Lara KA Perez A Alonso N Blanco-Vaca F Julve J . Novel insights into the role of HDL-associated sphingosine-1-phosphate in cardiometabolic diseases. Int J Mol Sci. (2019) 20:6273. doi: 10.3390/ijms20246273. PMID: 31842389 PMC6940915

[23] Bekpinar S Yenidunya G Gurdol F Unlucerci Y Aycan-Ustyol E Dinccag N . The effect of nephropathy on plasma sphingosine 1-phosphate concentrations in patients with type 2 diabetes. Clin Biochem. (2015) 48:1264–7. doi: 10.1016/j.clinbiochem.2015.08.001. PMID: 26255120

[24] Axler O Ahnstrom J Dahlback B . An ELISA for apolipoprotein M reveals a strong correlation to total cholesterol in human plasma. J Lipid Res. (2007) 48:1772–80. doi: 10.1194/jlr.m700113-jlr200. PMID: 17526892

[25] Christoffersen C Pedersen TX Gordts PL Roebroek AJ Dahlback B Nielsen LB . Opposing effects of apolipoprotein m on catabolism of apolipoprotein B-containing lipoproteins and atherosclerosis. Circ Res. (2010) 106:1624–34. doi: 10.1097/mol.0b013e328361f6ad. PMID: 20360257

[26] Hajny S Borup A Elsoe S Christoffersen C . Increased plasma apoM levels impair triglyceride turnover in mice. Biochim Biophys Acta Mol Cell Biol Lipids. (2021) 1866:158969. doi: 10.1016/j.bbalip.2021.158969. PMID: 34051379

[27] Stadler JT Borenich A Stattau Bisgaard L Bjergfelt SS Vijayakumar S Melholt L . ApoM and major adverse cardiovascular events in chronic kidney disease: A prospective cohort study. Arterioscler Thromb Vasc Biol. (2025) 45:496–505. doi: 10.1161/atvbaha.124.322367. PMID: 40047074 PMC11936471

[28] Ahnstrom J Axler O Jauhiainen M Salomaa V Havulinna AS Ehnholm C . Levels of apolipoprotein M are not associated with the risk of coronary heart disease in two independent case-control studies. J Lipid Res. (2008) 49:1912–7. doi: 10.1194/jlr.M700471-JLR200, PMID: 18490703

[29] Liu D Ji L Zhang D Tong X Pan B Liu P . Nonenzymatic glycation of high-density lipoprotein impairs its anti-inflammatory effects in innate immunity. Diabetes Metab Res Rev. (2012) 28:186–95. doi: 10.1016/b978-0-323-05659-5.00002-4. PMID: 21928330

[30] Brinck JW Thomas A Lauer E Jornayvaz FR Brulhart-Meynet MC Prost JC . Diabetes mellitus is associated with reduced high-density lipoprotein sphingosine-1-phosphate content and impaired high-density lipoprotein cardiac cell protection. Arterioscler Thromb Vasc Biol. (2016) 36:817–24. doi: 10.1161/atvbaha.115.307049. PMID: 26966278

[31] Elsoe S Ahnstrom J Christoffersen C Hoofnagle AN Plomgaard P Heinecke JW . Apolipoprotein M binds oxidized phospholipids and increases the antioxidant effect of HDL. Atherosclerosis. (2012) 221:91–7. doi: 10.1016/j.atherosclerosis.2011.11.031, PMID: 22204862

[32] Ahnstrom J Faber K Axler O Dahlback B . Hydrophobic ligand binding properties of the human lipocalin apolipoprotein M. J Lipid Res. (2007) 48:1754–62. doi: 10.1194/jlr.M700103-JLR200, PMID: 17525477

