# Supplementary material for: Relationship between apolipoprotein M levels and diabetic retinopathy in patients with type 2 diabetes mellitus
Source: Front Endocrinol (Lausanne). 2026 Apr 13;17:1809680. doi: 10.3389/fendo.2026.1809680 (PMC13111053; doi:10.3389/fendo.2026.1809680)
Supplement: Supplementary file 1 [file DataSheet1.pdf]

Relationship between apolipoprotein M levels and diabetic retinopathy in patients with type 2 diabetes mellitus

Jin Ook Chung[1], Seon-Young Park[2], Dong Jin Chung[1], Min Young Chung[1]

[1]Division of Endocrinology and Metabolism, Department of Internal Medicine, Chonnam National University Medical School, Gwangju, Republic of Korea
[2]Division of Gastroenterology and Hepatology, Department of Internal Medicine, Chonnam National University Medical School, Gwangju, Republic of Korea

Supplementary Table 1. Univariable logistic regression analysis for the association between plasma apoM and diabetic retinopathy

	OR (95% CI)	*P*-value
Age (years)	1.00 (0.99–1.02)	0.638
Sex (men)	0.97 (0.61–1.54)	0.891
Diabetes duration (years)†	7.74 (4.15–14.4)	<0.001
BMI (kg/m^2)	0.98 (0.93–1.04)	0.509
Hyperlipidemia (yes)	0.99 (0.54–1.80)	0.974
Hypertension (yes)	1.89 (1.15–3.12)	0.013
HbA$_{1c}$ (%)	1.03 (1.02–1.04)	<0.001
Total cholesterol (mmol/l)†	1.02 (0.90–1.15)	0.748
Triglyceride (mmol/l)†	1.69 (0.64–4.47)	0.290
HDL-cholesterol (mmol/l)†	0.47 (0.22–0.97)	0.041
LDL-cholesterol (mmol/l)†	0.88 (0.67–1.14)	0.328
hs-CRP (mg/L)†	1.49 (0.98–2.27)	0.065
ApoM (mg/L)†	1.72 (1.34–2.23)	<0.001
ApoA-I (g/L)†	0.93 (0.74–1.16)	0.497
ApoB (g/L)†	0.95 (0.76–1.20)	0.675
eGFR (ml·min^{-1}1.73m^{-2})	0.99 (0.98–0.998)	0.022
Urinary ACR (mg/g)†	2.86 (1.96–4.17)	<0.001
OHAs (yes)	1.98 (1.06–3.68)	0.032
Insulin (yes)	8.49 (4.62–15.58)	<0.001
Lipid-lowering agents (yes)	0.85 (0.50–1.44)	0.539

†Data were log$_{10}$-transformed before analysis.

OR, odds ratio; CI, confidence interval; BMI, body mass index; HbA$_{1c}$, glycated hemoglobin; HDL, high-density lipoprotein; LDL, low-density lipoprotein; hs-CRP, high-sensitivity C-reactive protein; apoM, apolipoprotein M; apoA-1, apolipoprotein A-1; apoB, apolipoprotein B; eGFR, estimated glomerular filtration rate; ACR, albumin excretion rate; OHAs, oral hypoglycemic agents

Supplementary Table 2. Collinearity diagnostics for the association between plasma apoM and diabetic retinopathy

	Variance Inflation Factor	
ApoM†	1.465	1.468
Age	1.899	1.873
Diabetes duration†	1.582	1.585
Hypertension (yes)	1.132	1.126
HbA_{1c}	1.168	1.169
HDL-cholesterol†	1.138	–
Hyperlipidemia (yes)	–	1.055
hs-CRP†	1.070	1.064
eGFR	2.062	1.983
Urinary ACR†	1.253	1.246
OHAs (yes)	1.285	1.221
Insulin (yes)	1.265	1.267

†Data were log_{10}-transformed before analysis.

apoM, apolipoprotein M; HbA_{1c}, glycated hemoglobin; HDL, high-density lipoprotein; hs-CRP, high-sensitivity C-reactive protein; eGFR, estimated glomerular filtration rate; ACR, albumin excretion rate; OHAs, oral hypoglycemic agents

Supplementary Table 3. Logistic regression models of the association between apoM levels and diabetic retinopathy in patients with T2DM

	Unadjusted model		Model 1		Model 2		Model 3	
	OR (95% CI)	*P*-value	OR (95% CI)	*P*-value	OR (95% CI)	*P*-value	OR (95% CI)	*P*-value
ApoM (mg/L)†	1.72 (1.34–2.23)	<0.001	1.89 (1.44–2.48)	<0.001	1.46 (1.08–1.98)	0.014	1.47 (1.04–2.08)	0.031
Tertile 1	1.00 (reference)		1.00 (reference)		1.00 (reference)		1.00 (reference)	
Tertile 2	1.92 (1.02–3.60)		1.94 (1.05–3.59)		1.84 (0.71-4.75)		1.79 (0.66–4.82)	
Tertile 3	1.99 (1.47–2.70)		2.34 (1.57–3.49)		2.24 (1.25–4.00)		2.20 (1.16–4.16)	
P for trend	<0.001		<0.001		0.015		0.026	

†Data were log_{10}-transformed before analysis.

Model 1: adjusted based on age, hypertension, hs-CRP†, and hyperlipidemia

Model 2: adjusted by model 1 plus HbA_{1c}, and diabetes duration†, and use of OHAs and insulin

Model 3: adjusted by model 2 plus urinary ACR† and eGFR

apoM, apolipoprotein M; HbA_{1c}, glycated hemoglobin; hs-CRP, high-sensitivity C-reactive protein; eGFR, estimated glomerular filtration rate; ACR, albumin excretion rate; OHAs, oral hypoglycemic agents

Supplementary Table 4. Sensitivity analyses adjusting for anti-hyperglycemic and lipid-lowering therapy

ApoM (mg/L)†	OR (95% CI)	*P*-value	% Change in OR
Model 1 (Base)	1.42 (1.02–1.98)	0.038	–
Model 2	1.49 (1.05–2.11)	0.027	4.9%
Model 3	1.46 (1.04–2.05)	0.028	2.8%

†Data were log_{10}-transformed before analysis.

Model 1: adjusted based on age, hs-CRP†, hypertension, HDL-cholesterol†, urinary ACR† and eGFR, HbA_{1c}, diabetes duration†

Model 2: adjusted by model 1 plus use of OHAs and insulin

Model 3: adjusted by model 1 plus use of lipid-lowering agents

apoM, apolipoprotein M; HbA_{1c}, glycated hemoglobin; HDL, high-density lipoprotein; hs-CRP, high-sensitivity C-reactive protein; eGFR, estimated glomerular filtration rate; ACR, albumin excretion rate; OHAs, oral hypoglycemic agents

Supplementary Table 5. Sensitivity analysis excluding patients with advanced renal impairment (n=308)

		Odds ratio	95% CI	*P*-value
ApoM (mg/L)†	Unadjusted model	1.64	1.23–2.18	0.001
	Model 1	1.51	1.07–2.13	0.019
	Model 2	1.54	1.07–2.23	0.021

†Data were $\log_{10}$-transformed before analysis. Regression estimates are shown for the subset of patients without overt macroalbuminuria (>300 mg/g) or severely reduced eGFR (< 30 $ml \cdot min^{-1} 1.73m^{-2}$).

Model 1: adjusted based on age, hypertension, hs-CRP†, HDL-cholesterol†, HbA_{1c}, diabetes duration†, and use of OHAs and insulin

Model 2: adjusted by model 1 plus urinary ACR† and eGFR

apoM, apolipoprotein M; HbA_{1c}, glycated hemoglobin; HDL, high-density lipoprotein; hs-CRP, high-sensitivity C-reactive protein; eGFR, estimated glomerular filtration rate; ACR, albumin excretion rate; OHAs, oral hypoglycemic agents
